# *Spartinivicinus ruber* gen. nov., sp. nov., a Novel Marine Gammaproteobacterium Producing Heptylprodigiosin and Cycloheptylprodigiosin as Major Red Pigments

**DOI:** 10.3389/fmicb.2020.02056

**Published:** 2020-08-28

**Authors:** Zhaobin Huang, Le Dong, Qiliang Lai, Jieqing Liu

**Affiliations:** ^1^College of Oceanology and Food Science, Quanzhou Normal University, Quanzhou, China; ^2^Fujian Province Key Laboratory for the Development of Bioactive Material from Marine Algae, Quanzhou Normal University, Quanzhou, China; ^3^Key Laboratory of Marine Genetic Resources, Third Institute of Oceanography, Ministry of Natural Resources, Xiamen, China; ^4^School of Biomedical Sciences, Huaqiao University, Quanzhou, China

**Keywords:** *Spartinivicinus ruber*, heptylprodigiosin, cycloheptylprodigiosin, nuclear magnetic resonance, bioactive assay, polyphasic taxonomy approach

## Abstract

The red pigment prodiginines are identified as bacterial secondary metabolites and display a wide range of bioactive properties. Here, a novel rose-red pigmented bacterium, designated strain S2-4-1H^T^, was isolated from coastal sediment of cordgrass *Spartina alterniflora*. Interestingly, it simultaneously produced heptylprodigiosin (C_22_H_29_N_3_O) and cycloheptylprodigiosin (C_22_H_27_N_3_O) as major red pigments, of which their chemical structures were established by liquid chromatography–mass spectrometry (LC–MS) and nuclear magnetic resonance (NMR). Bioactive assays revealed that both heptylprodigiosin and cycloheptylprodigiosin had antibacterial and antifungal activities, and notably, cycloheptylprodigiosin showed stronger bioactivity than heptylprodigiosin. The complete genome of strain S2-4-1H^T^ was determined to be 6,687,090 bp in length with a G + C content of 40.13 mol%, including a circular chromosome with a size of 6,361,125 bp and three plasmids with a size of 141,078, 102,423, and 82,464 bp, respectively. The biosynthetic gene cluster of two red pigments was predicted on a ∼41-kb gene fragment organized on the chromosome and displayed highly conserved features compared to several gammaproteobacterial species encoding the homologous genes. Finally, based on phenotypic, genotypic, and chemotaxonomic characteristics, strain S2-4-1H^T^ represented a novel genus-level species named *Spartinivicinus ruber* gen. nov., sp. nov. (type strain S2-4-1H^T^ = MCCC 1K03745^T^ = KCTC 72148^T^). Our study provided a novel bacterial source and novel prodigiosin analogs as promising pharmaceuticals in biotechnological application.

## Introduction

Prodiginines are a large family of red pigments bearing the linear tripyrrole core structure, which are produced by bacteria as secondary metabolites (Hu et al., 2016). As natural pigmented products, prodiginines have gained much more interests due to their antibacterial, antimalarial, algicidal, anticancer, immunosuppressive activities, and colorant for textile materials, etc. (Soliev et al., 2011; Stankovic et al., 2014; Yip et al., 2019).

Prodigiosin (2-methyl-3-amyl-6-methoxyprodigiosene), extracted from the ubiquitous bacterium *Serratia marcescens*, is the firstly characterized prodiginine with correct chemical structure, which is determined in the 1960s (Furstner, 2003; Williamson et al., 2006). Since then, prodigiosin (a pentyl chain at C-3 position) and its analogs have been widely found, especially in marine gammaproteobacteria, such as *Vibrio ruber* (Shieh et al., 2003), *Pseudoalteromonas rubra* (Gerber and Gauthier, 1979; Feher et al., 2008), *Zooshikella ganghwensis* (Yi et al., 2003; Lee et al., 2011), “*Zooshikella rubidus*” (Lee et al., 2011), *Zooshikella marina* (Ramaprasad et al., 2015), and *Hahella chejuensis* (Kim et al., 2007). Several actinomycetes, for instance, *Streptomyces coelicolor*, *Streptoverticillium rubrireticuli*, and *Saccharopolyspora* sp., can also produce prodigiosin analogs, but they have the aliphatic chain (an undecyl chain) at C-2 position, named undecylprodigiosin, which is different from prodigiosin on the chemical structure (Williamson et al., 2006).

Interestingly, several gammaproteobacterial species, such as *P. rubra* (Gerber and Gauthier, 1979) and *Z. marina* (Ramaprasad et al., 2015), are reported to simultaneously produce prodigiosin (C_20_H_25_N_3_O) and its carbocyclic isoform, cycloprodigiosin (C_20_H_23_N_3_O) as major components. Similarly, the actinomycete *S. coelicolor* A3(2) can also simultaneously produce undecylprodigiosin (C_25_H_35_N_3_O) and its carbocyclic isoform, butyl-meta-cyclo-heptylprodigiosin (streptorubin B, C_25_H_33_N_3_O) (Tsao et al., 1985) as major components. Heptylprodigiosin (a heptyl chain at C-3 position, C_22_H_29_N_3_O) has only been reported in a marine alphaproteobacterium, *Pseudovibrio denitrificans*, as a major component (Sertan-de Guzman et al., 2007) and in several marine gammaproteobacteria, *Z. marina* (Ramaprasad et al., 2015), *Z. rubidus* (Lee et al., 2011), and *H. chejuensis* (Kim et al., 2007), in minor amounts, whereas the chemical structure of its corresponding carbocyclic form is not reported so far.

The biosynthetic gene clusters of prodigiosin and undecylprodigiosin are fully characterized in *Serratia* sp. 39006 and *S. coelicolor* A3(2), which are organized in *pig* and *red* gene clusters, respectively (Williamson et al., 2006; Hu et al., 2016). The *pig* and *red* gene clusters exhibited distinguishable difference on the genetic organization by comparison of the homologous genes among *Serratia* sp. ATCC 39006, *Serratia marcescens* ATCC 274, *H. chejuensis* KCTC 2396^T^, and *S. coelicolor* A3(2) (Kim et al., 2006; Williamson et al., 2006). The biosynthesis of streptorubin B from undecylprodigiosin is accomplished by an enzyme named RedG in the *red* gene cluster of *S. coelicolor* A3(2). In contrast, the enzyme catalyzing the formation of cycloprodigiosin from prodigiosin was an alkylglycerol monooxygenase but was not related to RedG, which was experimentally verified in *P. rubra* DSM 6842^T^ (de Rond et al., 2017). Thus, it is proposed that the *pig* and *red* gene clusters possibly occurred in a divergent evolution (Williamson et al., 2006).

In this study, a novel strain designated S2-4-1H^T^ with distinct rose-red pigmented colony on agar plate was isolated from coastal sediment of cordgrass *Spartina alterniflora* in Quanzhou Bay, China. The chemical structures of red pigments were characterized, and bioactive assays against Gram-positive, Gram-negative bacteria, and fungi were carried out to evaluate the application potential as pharmaceuticals. The predicted functional genes were annotated by means of complete genome sequencing and gene annotation to find the biosynthetic gene clusters responsible for biosynthesis of the red pigments. Finally, we determined the taxonomic position of strain S2-4-1H^T^ using a polyphasic taxonomic approach.

## Materials and Methods

### Strain Isolation and Cultivation

The coastal surface sediment of cordgrass *S. alterniflora* in Quanzhou Bay (24°51 N, 118°40 E) was collected in November 2018. The samples were put into a sterile plastic bag and immediately taken to the laboratory. The sediment sample (0.2 g) was used for 10-fold serial dilution using sterile coastal seawater. An aliquot of 100 μl dilution (×10^4^) was spread onto a culture medium containing 18.7 g of Marine Broth 2216 (MB, BD, half of standard amount) and 15 g of Agar (BD, Difco) per 1 L. The agar plates were incubated at 28°C for 2 weeks in the dark. A distinct rose-red colored isolate designated S2-4-1H^T^ was picked and streaked onto fresh agar plates for isolation (Supplementary Figure S1). Cells were suspended in 20% glycerol (V/V) and stored at −80°C for further use. Strain S2-4-1H^T^ has been deposited in Marine Culture Collection of China (MCCC) and Korean Collection for Type Culture (KCTC) under the deposit number of MCCC 1K03745^T^ and KCTC 72148^T^, respectively.

### Phylogenetic Analysis Based on 16S rRNA Gene

Genomic DNA was extracted from the fresh cells of strain S2-4-1H^T^ using the Bacterial Genomic Extraction Kit (SBS, Co., Shanghai, China) following the manufacturer’s instructions. The strain was cultured in MB for 3 days of shaking at 160 rpm under 28°C. The 16S rRNA gene of strain S2-4-1H^T^ was amplified using bacterial primers Eubac27F and 1492R with *Ex* Taq (TaKaRa) in 50 μl of PCR system. The sequence was obtained from Sanger sequencing of the direct PCR product and quality controlled according to the chromatogram. The complete 16S rRNA gene sequence was obtained from the genome determined below and was compared to that of Sanger sequencing.

The close relatives were obtained from the EzBioCloud database (Yoon et al., 2017) and the NCBI nucleotide database^1^. These sequences were aligned using ClustalW with default parameters. The phylogenetic trees were constructed using two algorithms, neighbor-joining and maximum likelihood methods using MEGA 7.0 (Kumar et al., 2016). The best substitution model (GTR + G + I) for the maximum likelihood method was determined under the lowest BIC selection scores (Bayesian Information Criterion). The topologies of the phylogenetic trees were evaluated with bootstrapping of 1,000 replications for both methods.

### Red Pigment Extraction and Purification

Strain S2-4-1H^T^ grown on the agar plate was directly inoculated into a total of 6 L 0.8 × MB medium (ten 1,000-ml flasks with 600 ml liquid) and cultured for 48–52 h under 30°C and at 160 rpm for vigorous shaking to reach the stationary stage of the rose-colored liquid. Cell biomass was collected by centrifugation at 6,000 rpm for 10 min at room temperature, and the supernatant was discarded. The cells were lyophilized, and cellular pigments were extracted with dichloromethane (w/v = 1:30) from freeze-dried cells and subjected to sonication on ice for 20 min. The extractions were repeated three times, centrifugated, and evaporated to obtain the crude extract.

The crude extract was dissolved into ethyl acetate and loaded onto a thin-silica chromatography plate (10 cm × 20 cm). The red pigments were separated by using acetone and petroleum ether (v/v = 1.5:5) and then collected and eluted from the plate using ethyl acetate. The red pigments were dissolved into acetonitrile and analyzed by high-performance liquid chromatography (HPLC, Wates 2545, United States) to find the optimal separation condition. Pigment purification was performed using preparative HPLC (Waters 2695, United States) with 1.5 ml of liquid. The purification condition was Symmetry300^TM^ C18 Preo 5 μm column (19 mm × 15 cm) equipped with 10 ml/min flow rate with the 25% fluid A (0.08% trifluoroacetic acid with ddH_2_O) and 75% fluid B (0.08% trifluoroacetic acid with acetonitrile). Purification was further carried out repeatedly with the above same condition. Two major kinds of red pigments, named S-1 and S-2 were finally obtained.

### Structure Identification

The molecular weight of the two purified red pigments, S-1 and S-2, were analyzed by high-resolution mass spectrometry equipped with an electrospray ionization source (HRESIMS, APEX 7.0T FT-ICR-MS, ESI). Nuclear magnetic resonance (NMR) spectra of the pigments were measured on an instrument (BRUKER Ascend-400) for the operation at 400 MHz for ^1^H and 100 MHz for ^13^C NMR. Chemical structure of pigment S-1 was further performed on distortionless enhancement by polarization transfer (DEPT-135), heteronuclear multiple bond connectivity spectroscopy (HMBC), and heteronuclear single quantum correlation (HSQC) for further confirmation of C–H relationship.

### Bioactive Assays of Two Red Pigments

The antibacterial and antifungal activities of the two red pigments, S-1 and S-2, were tested against *Escherichia coli* JCM 1649^T^, *Staphylococcus aureus* CMCC (B) 26003^T^, *Bacillus subtilis* MCCC 1A00693^T^, and *Candida albicans* ATCC 10231 by disk diffusion method with different concentrations (12.5, 25, 50, 100, 500, and 1,000 μM). The tested strains were spread onto LB agar plates. The inhibition zone around the disk including disk diameter (6 mm) was measured for 48 h incubation at 37°C. All assays were performed in three replicates.

### Complete Genome Sequencing and Gene Annotation

Genomic DNA was extracted from fresh cells of strain S2-4-1H^T^ using FastDNA Spin Kit for Soil (MP, United States). DNA quality was controlled using 1% agarose electrophoresis and NanoDrop 2000 (Thermo, United States). The draft genome sequence was determined using the Illumina Hiseq platform (Shanghai Majorbio Bio-Pharm Technology Co., Ltd., Shanghai, China) according to the manufacturer’s instructions. Then, PacBio sequencing with one SMART cell was conducted from a 10-kb DNA fragment library using PacBio RSII sequencing platform according to the manufacturer’s instruction (MajorBio Co. Shanghai, China). Sequencing reads were assembled with both Hiseq short reads and PacBio long reads using the assemblers of Canu (Koren et al., 2017) and SPAdes v. 3.8.0 (Bankevich et al., 2012) to obtain the complete genome sequence, including the chromosome and plasmids.

The prediction of functional genes (coding genes, CDSs) of strain S2-4-1H^T^ was carried out using prodigal (Hyatt et al., 2010). The ribosomal RNA genes (rRNA operons) were predicted using RNAmmer (Lagesen et al., 2007). The tRNA genes were predicted using tRNAscan-SE v2.0 (Lowe and Eddy, 1997). Gene annotation was carried out by BLASTP search against nr database, Swiss-Prot database, the clusters of orthologous groups of proteins (COG) database, and KEGG database (Kanehisa et al., 2016) with an *e*-value cutoff of 1*e*^–5^ (Tatusov et al., 2001). The circular genome was drawn using Circos based on the above annotation (Krzywinski et al., 2009).

### Phenotypic, Genomic, and Chemotaxonomic Characterization

The type species *Z. ganghwensis* JC2044^T^ (=KCTC 12044^T^) was used as a reference strain according to the phylogenetic analysis. Gram-staining of strain S2-4-1H^T^ was performed using a Gram-staining kit (Hangzhou Tianhe Microorganism Reagent, Co., Ltd.). Cell morphology was observed using transmission electron microscopy (TEM, JEM-1230, and JEOL) after negative staining. Catalase and oxidase activities were tested by using 10% H_2_O_2_ solution and an oxidase reagent (BioMérieux, France), respectively. The growth temperatures of strain S2-4-1H^T^ and reference strain were determined by incubating the streaked plates of the strains at different temperatures (4, 10, 15, 20, 25, 28, 30, 35, 40, 45, and 50°C) for 1 week to observe their growth. The NaCl requirement and tolerance was determined using the basic component of MB (without NaCl) supplemented with various NaCl concentrations of 0, 0.5, 1, 2, 3, 4, 5, 6, 7, 8, 10, and 12% (w/v). Physiological activities and biochemical properties of strain S2-4-1H^T^ and the reference strain were tested using API ZYM, API 20NE, and API 20E kits according to the manufacturer’s instruction under the same conditions. The absorption spectra of the pigment extracted with acetone from strain S2-4-1H^T^ and reference strain JC2044^T^ were spectrophotometrically determined across a range of 200–900 nm using a spectrophotometer (TU-1950 Series UV-Vis, Persee).

Digital DNA–DNA hybridization (dDDH) estimate was calculated based on the whole genome sequences using the Genome-to-Genome Distance Calculator (GGDC 2.1) online service (Meier-Kolthoff et al., 2013). Average nucleotide identity (ANI) was calculated using OrthoANI computation on EzBioCloud tools (Lee et al., 2015). Average amino acid identity (AAI) was calculated using the genomic-wide identity suite (Rodriguez-R and Konstantinidis, 2014). The percentage of conserved proteins (POCP) was also used for genomic comparison with amino acid level (Qin et al., 2014).

The phylogenomic tree was constructed using MEGA 7.0 (Kumar et al., 2016) based on 43 conserved marker genes extracted with CheckM (Parks et al., 2015). The members affiliated with the order *Oceanospirillales* obtained from the genome portal in GenBank were selected as the reference genomes. The phylogenetic trees were constructed using two algorithms, neighbor-joining and maximum likelihood methods. The best substitution model (LG + G + I) for the maximum likelihood method was determined under the lowest BIC selection scores.

Polar lipids of strain S2-4-1H^T^ cultured for 48 h under 30°C and at 160 rpm for vigorous shaking were extracted using a chloroform/methanol system and analyzed by means of one- and two-dimensional TLC using Merck silica gel 60 F254 aluminum-backed thin-layer plates. Total lipids were detected by spraying the plate with 10% ethanolic molybdophosphoric acid and corresponding stain regents following our previous method (Huang et al., 2016). To measure and compare the fatty acid profile of strain S2-4-1H^T^ with reference strain, the two strains were cultured on agar plates at 28°C for 2 days. The cell biomass was collected, and cellular fatty acids were saponified, methylated and extracted, and then identified following the standard MIDI protocol (Sherlock Microbial Identification System, version 6B).

## Results

### Phylogenetic Analysis of Strain S2-4-1H^T^ Based on 16S rRNA Gene

In this study, a novel red-pigmented bacterial strain S2-4-1H^T^ was isolated from coastal sediment of cordgrass *S. alterniflora* (Supplementary Figure S1). The partial 16S rRNA gene (1403 bp) and complete 16S rRNA gene sequences (1529 bp) of strain S2-4-1H^T^ were obtained from Sanger sequencing and genome sequencing, respectively. The sequence similarity search against the EzBioCloud database showed that it had maximum similarities of 93.4, 93.4, 93.2, and 92.5% with *Z. ganghwensis* JC2044^T^, *Aestuariirhabdus litorea* GTF13^T^, *Z. marina* JC333^T^, and *Endozoicomonas euniceicola* EF212^T^, respectively, indicating that strain S2-4-1H^T^ represented a novel species, which was below the threshold for differentiating two species based on 16S rRNA gene sequence similarity of 98.65% (Kim et al., 2014).

**FIGURE 1:**
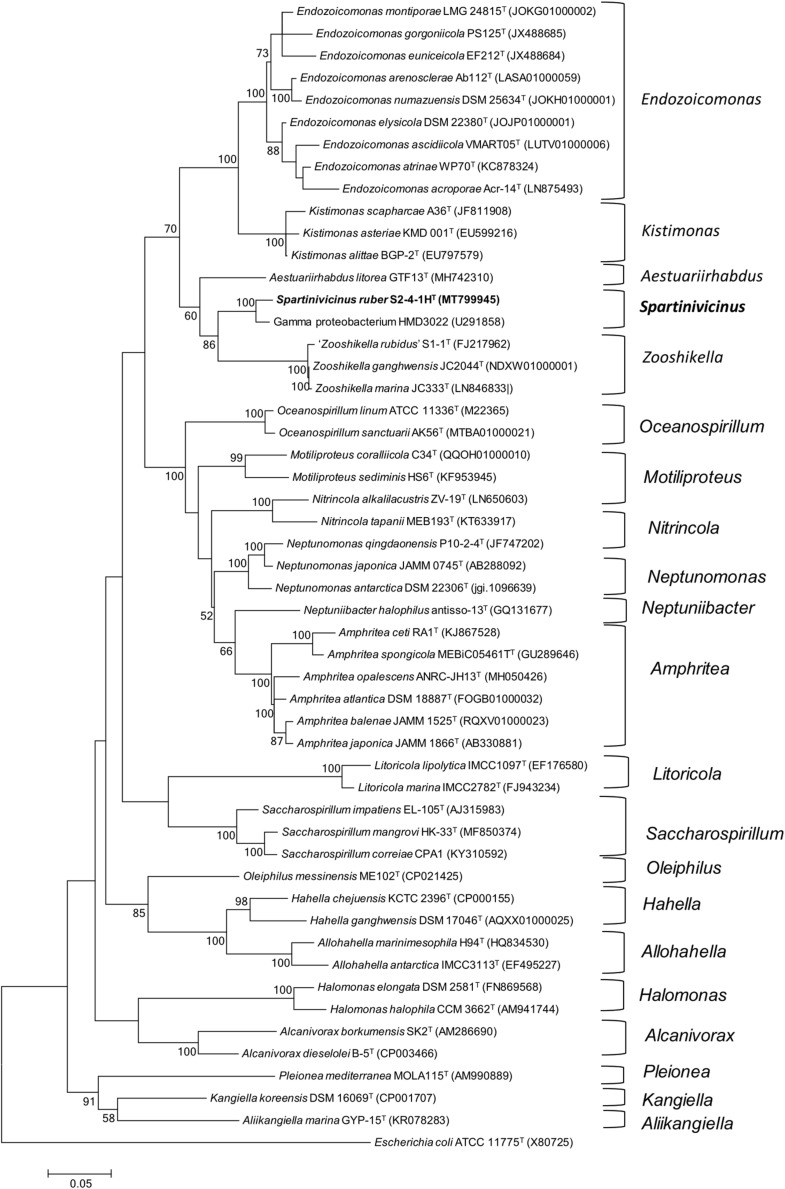
Phylogeny analysis of strain S2-4-1H^T^ compared to the members of the order *Oceanospirillales*. The maximum likelihood phylogenetic tree constructed based on 16S rRNA gene sequences showing the relationship of strain S2-4-1H^T^ with the closely related members within the order *Oceanospirillales*. Branch node values below 50% are not shown. *Spartinivicinus ruber* S2-4-1H^T^ are marked bold. *Escherichia coli* ATCC 11775^T^ (X80725) was selected as the outgroup. Bar, 0.05 represented the nucleotide substitution per position.

Phylogenetic analysis indicated that strain S2-4-1H^T^ belonged to the order *Oceanospirillales* and formed a novel and strongly supported cluster with a gammaproteobacterium strain HMD3022 (GenBank accession number: GU291858) sharing 98.4% sequence similarity, which was isolated from a solar saltern in Korea. It indicated that members of this novel cluster may be widespread in the coastal environment. Based on NJ (not shown) and ML phylogenetic analysis, this novel cluster containing strain S2-4-1H^T^ and strain HMD3022 should be considered as a novel species, which was closely neighbored with the members of the genus *Zooshikella*, including *Z. ganghwensis* JC2044^T^, *Z. marina* JC333^T^, and “*Z. rubidus*” S1-1 (Figure 1).

### Chemical Structure of Red Pigments Identified From Strain S2-4-1H^T^

The red pigments were extracted from the cell biomass and further purified using HPLC. Two major compounds designated S-1 and S-2 with a ratio of ∼1:1.5 were obtained at the retention time of 4.338 and 6.477 min, respectively (Supplementary Figure S2). The chemical structures of S-1 and S-2 were further determined with 1D-NMR and/or 2D-NMR spectra (DEPT-135, HMBC, and HSQC to confirm the C–H relationship of pigment S-1).

**FIGURE 2:**
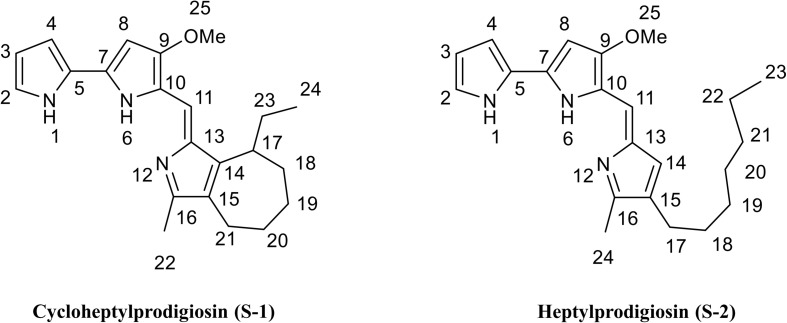
Chemical structure of cycloheptylprodigiosin (pigment S-1) and heptylprodigiosin (pigment S-2). MeO represents methoxy-group.

Compound S-1 was purple-red powder, and the molecular formula was determined by NMR and HRESIMS as C_22_H_27_N_3_O by [M + H]^+^ 350.2223 *m*/*z* (calculated 349.2154) (Supplementary Figure S3). The results of NMR (CDCl_3_) spectrum of compound S-1 are listed in Supplementary Table S1 and Supplementary Figure S4. Pigment S-1 exhibited three exchangeable protons [δ 12.48 (1H), 12.45 (1H), and 11.69 (1H)], five aromatic protons (δ 6.10–7.20), a vinylic proton (δ 6.90, s), a methoxyl proton (δ 4.00, s, 3H), an aromatic methyl proton (δ 2.37, s, 3H), plus seven cyclic alkyl containing two aromatic carbons [(δ 1.96, t, 2H), (δ 2.06, m, 1H), (δ 1.68, m, 4H), (δ 1.22, m, 2H)], linking an ethyl [(δ 1.25, m, 2H), (δ 0.86, t, 3H)]. The ^13^C NMR spectrum indicated that compound S-1 contained 22 carbons, which were also sorted out by the DEPT-135 spectrum with five CH_2_, one OCH_3_, two CH_3_, one CH of alkyl, five CH of vinyl, and eight quaternary C atoms (Supplementary Figure S4). On the basis of the ^1^JCH coupling, C2 (δ, 111.8) assigned to H2 (δ, 7.07), C3 (δ, 113.4) assigned to H3 (δ, 6.34), C4 (δ, 117.0) assigned to H4 (δ, 6.90), C8 (δ, 127.1) assigned to H8 (δ, 7.20), C11 (δ, 93.2) assigned to H11 (δ, 6.10), C19 (δ, 32.0) assigned to H19 (δ, 1.86), C8 (δ, 25.7) assigned to H8 (δ, 1.64), C22 (δ, 12.8) assigned to H22 (δ, 2.37), C24 (δ, 12.2) assigned to H24 (δ, 0.86), and C25 (δ, 58.8) assigned to H25 (δ, 4.00). On the basis of the ^2^JCH and ^3^JCH coupling, the methoxy (25-OMe, δ, 4.00) was assigned C9 (δ, 165.8), the methyl (22-Me, δ, 2.37) was assigned C16 (δ, 147.5) and C16 (δ, 124.4), the vinyl (11-C=, δ, 6.10) was assigned C9 (δ, 165.8) and C13 (δ, 122.2), the methyl (24-Me, δ, 0.86) was assigned C23 (δ, 25.4) and C17 (δ, 37.7), and the tertiary carbon (17- CH-, δ, 2.37) was assigned C14 (δ, 148.5), C23 (δ, 25.4), and C18 (δ, 28.8). These data indicated that there was a seven-member heterocycle with an ethyl. Thus, it can be inferred that compound S-1 was (Z)-8-ethyl-1-((3-methoxy-1H,1′H-[2,2′-bipyrrol]-5-yl)methylene)-3-methyl-1,4,5,6,7,8 hexahydrocyclohepta[c]pyrrole, which was named cycloheptylprodigiosin (Figure 2).

Compound S-2 was also purple-red powder, and the molecular formula was determined by NMR and HRESIMS as C_22_H_29_N_3_O by [M + H]^+^ 352.23131 *m*/*z* (calculated 351.2311) (Supplementary Figure S3). The results of NMR (CDCl_3_) spectrum of compound S-2 are listed in Supplementary Table S2 and Supplementary Figure S5. Pigment S-2 exhibited three exchangeable protons [δ 12.57 (1H), 12.57 (1H), and 12.00 (1H)], five aromatic protons (δ 6.08-7.24), a vinylic proton (δ 6.93, s), a methoxyl proton (δ 3.99, s, 3H), an aromatic methyl proton (δ 2.39, s, 3H), plus the protons of seven alkyl groups [(δ 2.32, t, 2H), (δ 1.25–1.30, m, 10H), (δ 0.88, t, 3H)], linking an aromatic group. The ^13^C NMR spectrum of compound S-2 indicated that it contained 22 carbons (δ 166.3, 148.6, 146.5, 129.3, 128.6, 128.0, 125.8, 122, 121.3, 118.0, 116.2, 112.0, 93.5, 58.8, 32.0, 30.3, 29.4, 29.3, 25.5, 22.8, 14.2, 12.2), which were also sorted out by six CH_2_, one OCH_3_, two CH_3_, and 13 C of vinyl (Supplementary Figure S5). This compound was 4-methoxy-5-[(4-heptyl-5-methyl-2H-pyrrol-2-ylidene) methyl]-1H,1′H-2,2′-bi-pyrrole), named heptylprodigiosin (16-methyl-15-heptyl-prodiginine) (Figure 2).

### Bioactive Assays Against Microorganisms

**FIGURE 3:**
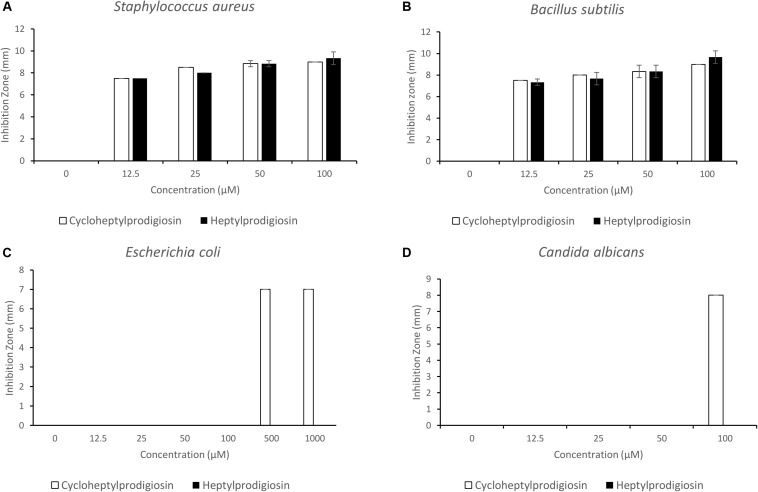
Bioactive assays of heptylprodigiosin and cycloheptylprodigiosin against *Staphylococcus aureus* CMCC (B) 26003^T^ **(A)**, *Bacillus subtilis* MCCC 1A00693^T^ **(B)**, *Escherichia coli* JCM 1649^T^ **(C)**, and *Candida albicans* ATCC 10231 **(D)**. The red pigments were dissolved in 0.1% DMSO. All assays were carried out in triplicate, and error bars represent standard deviation of the average value.

The antibacterial and antifungal activities of two red pigments purified from strain S2-4-1H^T^ were determined in this study. The results showed that both heptylprodigiosin and cycloheptylprodigiosin showed antibacterial activities against *E. coli* JCM 1649^T^, *S. aureus* CMCC (B) 26003^T^, and *B. subtilis* MCCC 1A00693^T^ and antifungal activity against *C. albicans* ATCC 10231. Interestingly, heptylprodigiosin and cycloheptylprodigiosin demonstrated similar bioactivities against Gram-positive bacteria, *S. aureus* CMCC (B) 26003^T^ and *B. subtilis* MCCC 1A00693^T^, at concentrations of 12.5, 25, 50, and 100 μM. Cycloheptylprodigiosin had stronger inhibition activity than heptylprodigiosin against *E. coli* JCM 1649^T^ and *C. albicans* ATCC 10231 (Figure 3). For instance, 1 mM cycloheptylprodigiosin can inhibit the growth of *E. coli* JCM 1649^T^, while heptylprodigiosin cannot under the same concentration.

### Genomic Features of Strain S2-4-1H^T^ and Predicted Biosynthetic Gene Clusters

**FIGURE 4:**
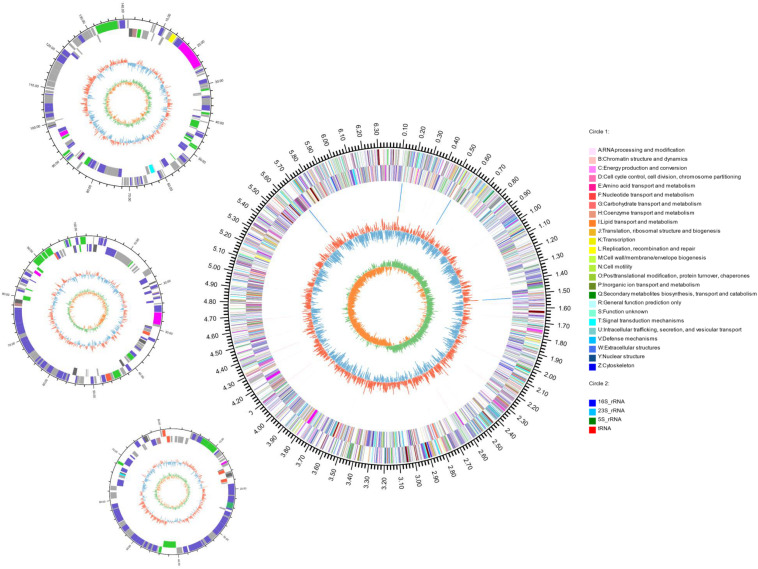
Circular representation of the complete genome of *S. ruber* S2-4-1H^T^. The features with circles were numbered from the outermost (first circle) to the innermost (seventh circle). The first circle represents the genome size, and second and third circles are COG functional categories of CDSs on forward and reverse strand; the fourth circle are tRNA and rRNA loci; the fifth circle is GC content; the sixth circle is GC skew. The circular representation was computed and drawn by the Circos program.

The complete genome of strain S2-4-1H^T^ was obtained by combining the assembly of both Illumina paired end reads and PacBio long reads. A total of 5,055,832 clean reads and 426,833 clean single reads were obtained from Illumina Hiseq. A total of 424,411 clean long reads were obtained with the largest reads of 75,662 bp retrieved from one SMART cell of PacBio RSII sequencing (average length of 5583.83 bp). The complete genome size of strain S2-4-1H^T^ was 6,687,090 bp with a G + C content of 40.13 mol%, including a circular chromosome with a size of 6,361,125 bp with a G + C content of 40.09 mol%, and three plasmids with a size of 141,078 bp (Plasmid A) with a G + C of 40.37 mol%, 102,423 bp (Plasmid B) with a G + C of 40.46 mol%, and 82,464 bp (Plasmid C) with a G + C of 41.98 mol%, respectively (Figure 4 and Supplementary Table S3).

Through gene annotation, we found that the complete genome encoded 6148 CDSs and had four copies of *rrn* operon (16S-23S-5S rRNA genes) with 100% 16S rRNA gene sequence (1529 bp) identity of each other and 65 tRNA genes; gene density reached 0.98 (Figure 4 and Supplementary Table S3). The genome contained 5746 CDSs, and the three plasmids A, B, and C contained 161, 136, and 105 ORFs, respectively (Supplementary Table S3).

**FIGURE 5:**
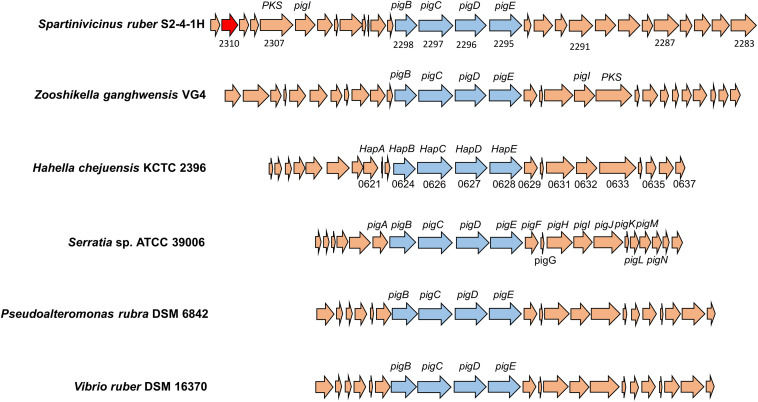
The organization of biosynthetic gene clusters of red pigments of *S. ruber* S2-4-1H^T^ compared to other gammaproteobacterial members. The gene number of *H. chejuensis* KCTC 2396^T^ was annotated with *hap* genes, which are already reported as homology of *pig* genes in *Serratia* sp. 39006. Gene annotation: 2285, FAD/NAD(P)-binding oxidoreductase; 2290, dehydrogenase; 2295, putative aminotransferase pigE; 2296, conserved hypothetical protein pigD; 2297, phosphoenolpyruvate synthase; 2300, putative acyl-CoA dehydrogenase pigA; 2302, acyl carrier protein; 2303, 8-amino-7-oxononanoate synthase; 2304, RedY protein; 2306, putative L-prolyl-AMP ligase pigI; 2308, methyltransferase type 12; 2310, hypothetical protein PRUB_00680 (sharing amino acid similarity of 48.1%); 2311, LuxR family transcriptional regulator.

The biosynthetic gene cluster for heptylprodigiosin and cycloheptylprodigiosin was predicted through functional gene annotation and comparative genes with close relatives. The total sequence length of this gene cluster was ∼41 kb and contained 29 genes in the chromosome (Figure 5). Through comparative analysis of the biosynthetic genes of strain S2-4-1H^T^ and those of other close related gammaproteobacterial members, including *Z. ganghwensis* VG4, *H. chejuensis* KCTC 2396^T^, *Serratia* sp. ATCC 39006, *P. rubra* DSM 6842, and *V. ruber* DSM 16370, we found that the biosynthetic gene cluster of strain S2-4-1H^T^ was larger than the other close relatives and they demonstrated more similar structure to *Z. ganghwensis* VG4 (Rehman et al., 2018). Notably, the homolog genes of adjacent *pigB*, *pigC*, *pigD*, and *pigE* were more highly conserved in *S. ruber* S2-4-1H^T^ compared to the close relatives (Figure 5). However, more than half of the genes could not be annotated to known function, indicating the novelty of gene participating the biosynthesis of the red pigments. In addition, we found that gene2310 shared 48.1% sequence similarity with gene PRUB680 affiliating with alkylglycerol monooxygenase in *P. rubra* DSM 6842, which was experimentally confirmed to convert the linear to carbocyclic congener of prodigiosin (de Rond et al., 2017).

### Phenotypic, Genomic, and Chemotaxonomic Characteristics of Strain S2-4-1H^T^

Colonies of strain S2-4-1H^T^ cultured at 30°C on MB agar plate were round, rose-red color (Supplementary Figure S1), which can distinctly differentiate the colony morphologies of *Z. ganghwensis* JC2044^T^ of red pigment with a metallic green sheen and *A. litorea* GTF13^T^ that is opaque and smooth (Table 1). A maximum peak at 532–535 nm of the absorption spectrum of the pigment extracted with acetone from strain S2-4-1H^T^ was determined, which showed similar profile to the spectrum pattern of *Z. ganghwensis* strain JC2044^T^ with a maximum peak at 532 nm (Supplementary Figure S6).

**TABLE 1:** Differential characteristics of strain S2-4-1H^T^ compared to closely related members of the genera *Zooshikella* and *Aestuariirhabdus* (1) Strain S2-4-1H^T^; (2) *Z. ganghwensis* JC2044^T^; (3) *Z. marina* JC333^T^; (4) *Aestuariirhabdus litorea* GTF13^T^.

**Characteristics**	**1**	**2**	**3^b^**	**4^c^**
Colony on agar plate	Rose-red without a metallic color	Red with a metallic sheen^a^	Red	Opaque
Cell size (μm)	2.0–2.3 × 0.9	1.5–2.5 × 0.7–0.9^a^	0.6–0.8 × 1.8–3.5	ND
Growth temperature (optimum, °C)	15–40 (35)	15–45 (35–40)	15–40 (30)	20–37 (30)
NaCl requirement (optimum,%)	0–7 (2)	1–7 (3–4)^a^	0–8 (2)	1–6 (2)
Cystine arylamidase	+	w	−	+
Acid phosphatase	−	+	+	+
NaphtholAS-BI-phosphohydrolase	w	+	+	+
Arginine dihydrolase	−	+	+	+
Utilization of citrate	+	−	−	−
Hydrolysis of gelatin	−	+	−	+
Major fatty acids (>10%)	SF 3 (C_16__:__1_ ω7*c* and/or C_16__:__1_ ω6*c*), C_16__:__0_, C_10__:__0_ 3-OH, C_12__:__0_ 3-OH	SF 3 (C_16__:__1_ ω7*c* and/or C_16__:__1_ ω6*c*), C_16__:__0_	SF 3 (C_16__:__1_ ω7*c* and/or C_16__:__1_ ω6*c*), C_16__:__0_, SF 8 (C_18__:__1_ ω7*c*)	SF 3 (C_16__:__1_ω7*c* and/or C_16__:__1_ω6*c*), C_16__:__0_, SF 8 (C_18__:__1_ ω7*c* and/or C_18__:__1_ ω6*c*)
Major polar lipids	PE, PG, DPG, PL	PE, PG, DPG^b^	PE, PG, DPG	PE, PL
Genome size (Mb)	6.69	5.80	ND	3.66
DNA G + C content (%)	40.1	41.0	40.2	59.8

*Data were obtained from this study unless indicated. SF, summed feature; PE, phosphatidylethanolamine; PG, phosphatidylglycerol; DPG, diphosphatidylglycerol; PL, phospholipid; ND, no data. ^*a*^Data were taken from Yi et al. (2003). ^*b*^Data were taken from Ramaprasad et al. (2015). ^*c*^Data were taken from Khan et al. (2020).*

The cells of strain S2-4-1H^T^ were Gram-stain-negative, rod-shaped, 2.0–2.3 μm long, and 0.9 μm wide, and no flagellum (Supplementary Figure S7). Catalase activity and oxidase activity were found to be positive. Growth was observed at 15–40°C with the optimum at 35°C, which was different from the close relative *Z. ganghwensis* JC2044^T^ growing from 15 to 45°C. No growth of strain S2-4-1H^T^ occurred at 10, 45, and 50°C. NaCl requirement was determined to be 0–7% NaCl (w/v) with the optimum of 2%, which was also different from *Z. ganghwensis* JC2044^T^ and *A. litorea* GTF13^T^ (Table 1).

Strain S2-4-1H^T^ shows positive results for alkaline phosphatase, esterase (C4), esterase lipase (C8), leucine arylamidase, valine arylamidase, cystine arylamidase; weak positive for lipase (C14) and naphthol-AS-BI-phosphohydrolase. It can utilize citrate. Voges–Proskauer reaction is positive, which is similar to that of *Z. ganghwensis* JC2044^T^. Hydrolysis of gelatin and arginine dihydrolase are negative for strain S2-4-1H^T^, which were different from *Z. ganghwensis* JC2044^T^ of positive reaction (Table 1). It can weakly utilize D-glucose as sole carbon source.

Digital DNA–DNA hybridization value compared between S2-4-1H^T^ and *Z. ganghwensis* JC2044^T^ was 24.60%, which was also below the threshold values of bacterial species delineation. The average nucleotide identity (ANI) value of strain S2-4-1H^T^ compared to *Z. ganghwensis* JC2044^T^ and *A. litorea* GTF13^T^ was estimated to be 69.0 and 66.8%, respectively, which was below the standard criteria of 95.0–96.0% for delineation of the same species. These data indicated that strain S2-4-1H^T^ represented a novel species. Since ANI value with <70% is not confident, the average AAI and POCP were calculated. AAI of strain S2-4-1H^T^ and *Z. ganghwensis* JC2044^T^ was 58.60%, which was below the new genus value of 65% (Konstantinidis et al., 2017). The POCP values of strain S2-4-1H^T^ compared to *Z. ganghwensis* JC2044^T^ and *A. litorea* GTF13^T^ were 51.39 and 36.42%, respectively. Notably, strain S2-4-1H^T^ had a much larger genome size and much lower G + C content than *A. litorea* GTF13^T^ (Table 1), which make them different.

Phylogenomic construction based on conserved marker genes also placed strain S2-4-1H^T^ into a novel clade, which was closely related to the members of the genus *Zooshikella* within the order *Oceanospirillales*. Notably, it was phylogenetically distinct from the members within the family *Hahellaceae* consisting two species, *H. chejuensis* KCTC 2396^T^ and *H. ganghwensis* DSM 17046^T^ (Supplementary Figure S8).

Combined with the phenotypic characteristics and the prodiginines produced, we proposed that S2-4-1H^T^ should represent a novel genus-level species, which is different from the genera *Zooshikella* and *Aestuariirhabdus*. Thus, this novel species was named *Spartinivicinus ruber.*

The polar lipids of strain S2-4-1H^T^ are identified as phosphatidylethanolamine (PE), phosphatidylglycerol (PG), diphosphatidylglycerol (DPG), aminophospholipid (APL), three unidentified phospholipids (PL), and two unidentified lipids (L) (Supplementary Figure S9). PE, PG, and DPG were also identified in the close relatives, *Z*. *ganghwensis* JC2044^T^ and *Z. marina* JC333^T^ (Ramaprasad et al., 2015), but showed different numbers of APL and L with strain S2-4-1H^T^. The major fatty acids (>10%) of strain S2-4-1H^T^ were summed feature 3 (C_16__:__1_ω7*c* and/or C_16__:__1_ω6*c*) (29.2%), C_16__:__0_ (21.3%), C_10__:__0_ 3-OH (13.7%), and C_12__:__0_ 3-OH (11.3%), which had a different pattern with *Z. ganghwensis* JC2044^T^ (Supplementary Table S4).

## Discussion

Since prodigiosin is firstly confirmed by chemical synthesis in 1960–1962 (Williamson et al., 2006), several structural classes of prodigiosin analogs are identified by using NMR spectroscopic data, for instance, cycloprodigiosin, undecylprodigiosin, streptorubin B, cyclononylprodigiosin (Williamson et al., 2006; Hu et al., 2016), and heptylprodigiosin (Sertan-de Guzman et al., 2007). Here, we characterized a novel prodigiosin analog named cycloheptylprodigiosin (Figure 2), of which chemical structure was determined by means of mass spectra and NMR. Notably, cycloheptylprodigiosin (C_22_H_27_N_3_O) demonstrated a novel feature of the cyclization of heptylprodigiosin (Figure 2). This is the first study of a bacterium that simultaneously produced heptylprodigiosin and cycloheptylprodigiosin as two major metabolites.

Prodigiosins are attracting more interest due to the antimicrobial activities (Stankovic et al., 2014; Yip et al., 2019). It is reported that the cycloprodigiosin was more active against several kinds of bacteria than the linear form, prodigiosin (Lee et al., 2011). Our results also demonstrated that cycloheptylprodigiosin had better bioactivity than heptylprodigiosin under the same concentration, especially against the Gram-negative *E. coli* and fungus *C. albicans* (Figure 3). The antibacterial activity against Gram-positive bacteria showed a similar pattern of inhibition of different concentration. The mechanism of different response to the tested bacteria will be studied further.

The production of prodigiosin in *S. marcescens* ATCC 274 and *Serratia* sp. ATCC 39006 (Harris et al., 2004) and *H. chejuensis* KCTC 2396 (Kim et al., 2006) is well known to be untaken by the *pig* gene cluster and *hap* gene cluster, respectively. Through comparative genomic analysis, there is a biosynthetic gene cluster in *S. ruber* S2-4-1H^T^ exhibiting highly conserved gene structure with *pig* and *hap* clusters, which could be explained for the production of the two red pigments, cycloheptylprodigiosin and heptylprodigiosin (Figure 5). A homolog of the alkylglycerol monooxygenase was found in *S. ruber* S2-4-1H^T^, which was presumed to perform the oxidation cyclization of heptylprodigiosin. However, due to the majority of unknown genes, the biosynthetic pathway of the two pigments in *S. ruber* S2-4-1H^T^ will be studied further.

The polyphasic taxonomy approaches including phenotypic, phylogenetic, and genomic, and chemical component analysis indicated that strain S2-4-1H^T^ represented a novel species within a novel genus named *Spartinivicinus*, closely related with *Zooshikella* (Figure 1 and Supplementary Figure S8). Currently, *Zooshikella* was classified into the family *Hahellaceae* in the order *Oceanospirillales* (EzBioCloud Database). Phylogenetic analysis based on 16S rRNA gene (Figure 1) and conserved marker genes (Supplementary Figure S8) did not place *Zooshikella* in the family *Hahellaceae*, although the type strain *H. chejuensis* KCTC 2396^T^ was found to produce prodigiosin (Kim et al., 2007). The gene organization of the prodigiosin biosynthetic gene cluster in *Spartinivicinus* is more similar to that of *Zooshikella* than *Hahella*, which also supported the idea that *Spartinivicinus* and *Zooshikella* are closely related (Figure 5).

In conclusion, we reported that a novel bacterium named *S. ruber* gen. nov., sp. nov. can simultaneously produce heptylprodigiosin (C_22_H_29_N_3_O) and cycloheptylprodigiosin (C_22_H_27_N_3_O) as determined by means of mass spectrometry and nuclear magnetic resonance. The new feature of the cyclization of heptylprodigiosin was reported. Bioactive assays showed that heptylprodigiosin and cycloheptylprodigiosin had antibacterial and antifungal activities. Their production can be explained by the presence of a conserved biosynthetic gene cluster organized on the chromosome and displayed highly conserved features compared to several gammaproteobacterial species encoding the homologous genes. Our study provided a new producer of prodiginine, which further expends the resource of bacterial secondary metabolites and provides a new model organism for prodiginine study.

### Description of *Spartinivicinus* gen. nov.

*Spartinivicinus ruber* (Spar.ti.nivi’ci.nus, N.L. fam. n. *Spartina* a seagrass genus; L. masc. n. *vicinus* a neighbor; N.L. masc. n. *Spartinivicinus*, a neighbor of *Spartina*).

Cells are Gram-negative and aerobic. Colonies on MB agar plate are circular, rose-red pigmented. Catalase activity and oxidase activity were positive. Heptylprodigiosin and Cyclcoheptylprodigiosin are produced. The predominant fatty acids are summed feature 3 (C_16__:__1_ω7*c* and/or C_16__:__1_ω6*c*), C_16__:__0_ C_10__:__0_ 3-OH, and C_12__:__0_ 3-OH. Polar lipid mainly consisted of phosphatidylethanolamine, phosphatidylglycerol, and diphosphatidylglycerol. The G + C content was ∼40.1 mol%. The type species is *S. ruber*.

### Description of *S. ruber* sp. nov.

*Spartinivicinus ruber* (ru’ber. L. masc. adj. *ruber* red).

The species exhibits the following properties in addition to those described for the genus. Cells are rod-shaped, 2.0–2.3 μm long and 0.9 μm wide, and no flagellum. Growth is observed at 15–40°C with the optimum at 35°C. No growth is observed at 45°C and above. NaCl requirement occurs to be 0–7% NaCl (w/v) with the optimum of 2%. Alkaline phosphatase, esterase (C4), esterase lipase (C8), leucine arylamidase, valine arylamidase, and cystine arylamidase are positive. Lipase (C14) and naphthol-AS-BI-phosphohydrolase are weak positive. It can utilize citrate. Voges–Proskauer reaction is positive. Hydrolysis of gelatin and arginine dihydrolase is negative. It can weakly utilize D-glucose as sole carbon source. The DNA G + C content of type strain is 40.1 mol%.

The GenBank accession numbers of the 16S rRNA gene sequence of strain S2-4-1H^T^ are MN808565 and MT799945. The complete genome sequence of strain S2-4-1H^T^ has been deposited at GenBank database under accession number CP048878-CP048881.

The type strain S2-4-1H^T^ (= MCCC 1K03745^T^ = KCTC 72148^T^) was isolated from sediment of cordgrass *S. alterniflora* collected from Quanzhou Bay, Fujian Province, China.

## Data Availability Statement

The datasets generated in this study can be found in online repositories. The names of the repository/repositories and accession number(s) can be found below: https://www.ncbi.nlm.nih.gov/genbank/, MN808565, MT799945 and https://www.ncbi.nlm.nih.gov/genbank/, CP048878–CP048881.

## Author Contributions

ZH conceived and designed the research. ZH, LD, and QL conducted the experiments. ZH, LD, and JL analyzed the data. ZH and LD wrote the manuscript. All authors read and approved the manuscript.

## Conflict of Interest

The authors declare that the research was conducted in the absence of any commercial or financial relationships that could be construed as a potential conflict of interest.

## Footnotes

1 https://blast.ncbi.nlm.nih.gov/Blast.cgi
