# Supplementary material for: Spartinivicinus ruber gen. nov., sp. nov., a Novel Marine Gammaproteobacterium Producing Heptylprodigiosin and Cycloheptylprodigiosin as Major Red Pigments
Source: Front Microbiol. 2020 Aug 28;11:2056. doi: 10.3389/fmicb.2020.02056 (PMC7484904; doi:10.3389/fmicb.2020.02056)
Supplement: Supplementary file 1 [file Data_Sheet_1.docx]

**Supplementary Tables.**

**Supplementary Table 1**. 1D- and 2D-NMR data (^1^H NMR, ^13^C NMR, C-DEPT135, HMBC and HSQC) of compound S-1 (CDCl_3_, 400MHz, all δ values in ppm and J values in Hz)

| Position | ^1^H NMR | ^13^C NMR | C-DEPT135 | HSQC | HMBC (^2^J_CH,_^3^J_CH_) |
| --- | --- | --- | --- | --- | --- |
| 1 | 12.48 s |  |  |  |  |
| 2 | 7.07 d | 111.8 | 111.7 | C2 | C5 |
| 3 | 6.34 m | 113.4 | 113.3 | C3 |  |
| 4 | 6.90 s | 117.0 | 116.9 | C4 |  |
| 5 |  | 146.4 |  |  |  |
| 6 | 12.45 s |  |  |  |  |
| 7 |  | 127.3 |  |  |  |
| 8 | 7.20 s | 127.1 | 127.0 | C8 |  |
| 9 |  | 165.8 |  |  |  |
| 10 |  | 120.5 |  |  |  |
| 11 | 6.10 (d, 2.2) | 93.2 | 93.5 | C11 | C9, C13 |
| 12 | 11.69 |  |  |  |  |
| 13 |  | 122.2 |  |  |  |
| 14 |  | 148.5 |  |  |  |
| 15 |  | 124.4 |  |  |  |
| 16 |  | 147.5 |  |  |  |
| 17 | 1.96 m | 37.7 | 37.6 |  | C23, C14, C18 |
| 18 | 1.64 m | 28.8 |  |  |  |
| 19 | 1.86 m | 32.0 | 31.9 | C19 |  |
| 20 | 1.64 m | 25.7 | 25.6 | C18 |  |
| 21 | 2.06 t | 25.7 | 25.6 |  |  |
| 22-Me | 2.37 s | 12.8 | 12.7 | C22 | C15, C16 |
| 23 | 1.25 m | 25.4 | 25.3 |  |  |
| 24 | 0.86 t | 12.2 | 12.1 | C24 | C23, C17 |
| 25-OMe | 4.00 s | 58.8 | 58.7 | C25 | C9 |

**Supplementary Table 2**. 1D-NMR data (^1^H NMR, ^13^C NMR) of compound S-2 (CDCl_3_, 400MHz).

| Position | ^1^H-NMR | ^13^C-NMR |
| --- | --- | --- |
| 1 | 12.57 s |  |
| 2 | 6.95 s | 125.8 |
| 3 | 6.36 m | 116.2 |
| 4 | 6.08 d | 112.1 |
| 5 |  | 128.0 |
| 6 | 12.50 s |  |
| 7 |  | 128.6 |
| 8 | 6.93 s | 121.3 |
| 9 |  | 166.3 |
| 10 |  | 112.0 |
| 11 | 6.69 s | 93.5 |
| 12 | 12.00 s |  |
| 13 |  | 118.0 |
| 14 | 7.24 s | 129.3 |
| 15 |  | 146.5 |
| 16 |  | 148.3 |
| 17 | 2.36 t | 29.3 |
| 18 | 1.25-1.30 m | 25.5 |
| 19 | 1.25-1.30 m | 29.4 |
| 20 | 1.25-1.30 m | 30.3 |
| 21 | 1.25-1.30 m | 32.0 |
| 22 | 1.25-1.30 m | 22.8 |
| 23 | 0.88 t | 12.2 |
| 24-Me | 2.39 s | 14.2 |
| 25-OMe | 3.99 s | 58.8 |

**Supplementary Table 3**. Genomic features of *Spartinivicinus ruber* S2-4-1H^T^.

| Genomic features | Chromosome | Plasmid A | Plasmid B | Plasmid C |
| --- | --- | --- | --- | --- |
| Genomic size (bp) | 6,361,125 | 141,078 | 102,423 | 82,464 |
| G+C content (%) | 40.09 | 40.37 | 40.46 | 41.98 |
| CDS^a^ | 5,746 | 161 | 136 | 105 |
| Hypothetical proteins^b^ | 2,662 | 120 | 85 | 68 |
| No hits found^b^ | 485 | 25 | 26 | 14 |
| tRNA | 65 | 0 | 0 | 0 |
| rRNA (16S-23S-5S) | 4 | 0 | 0 | 0 |

a. CDS means the number of predicted protein-coding sequences.

b. Counts based on blast against nr protein database (e-value of 1e-5).

**Supplementary Table 4**. Cellular fatty acids profiles of strain S2-4-1H^T^ compared to the closely related member, *Zooshikella ganghwensis* JC2044^T^.

1. Strain S2-4-1H^T^; 2. *Zooshikella ganghwensis* JC2044^T^.

tr, trace (<1%), -, not detected.

| Fatty acids | 1 | 2 |
| --- | --- | --- |
| Saturated |  |  |
| C_10:0_ | 4.3 | 3.5 |
| C_12:0_ | - | 1.0 |
| C_14:0_ | 1.1 | 7.8 |
| C_16:0_ | 21.3 | 23.4 |
| Hydroxyl |  |  |
| C_8:0_ 3-OH | 1.3 | tr |
| C_10:0_ 3-OH | 13.7 | 4.2 |
| C_12:1_ 3-OH | 5.8 | tr |
| C_12:0_ 3-OH | 11.3 | 4.3 |
| Unsaturated |  |  |
| C_16:1_ *ω*5*c* | tr | 1.9 |
| Summed feature 2† | 1.2 | tr |
| Summed feature 3† | 29.2 | 40.4 |
| Summed feature 8† | 7.1 | 9.2 |

†Summed features are groups of two or three fatty acids that cannot be separated by GLC using the MIDI system. Summed feature 2 comprised C_12:0_ aldehyde/unknown 10.928, summed feature 3 comprised C_16:1_*ω*7*c* and/or C_16:1_*ω*6*c*, and summed feature 8 comprised C_18:1_ *ω*7*c* and/or C_18:1_ *ω*6*c*.

**Supplementary Figures.**

**
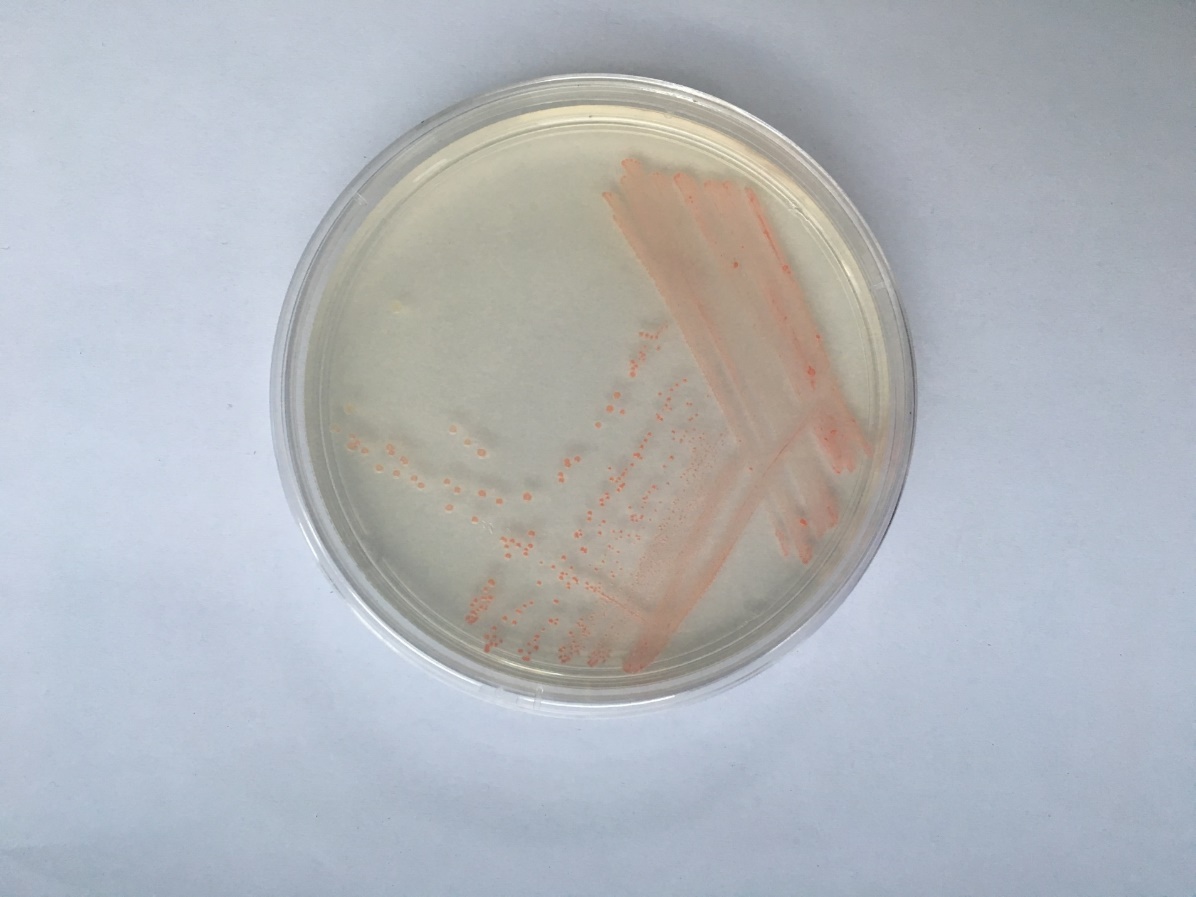
**

**Figure S1.** Colony morphology of strain S2-4-1H^T^ on MB agar plate cultured at 30^o^C for 3 days.


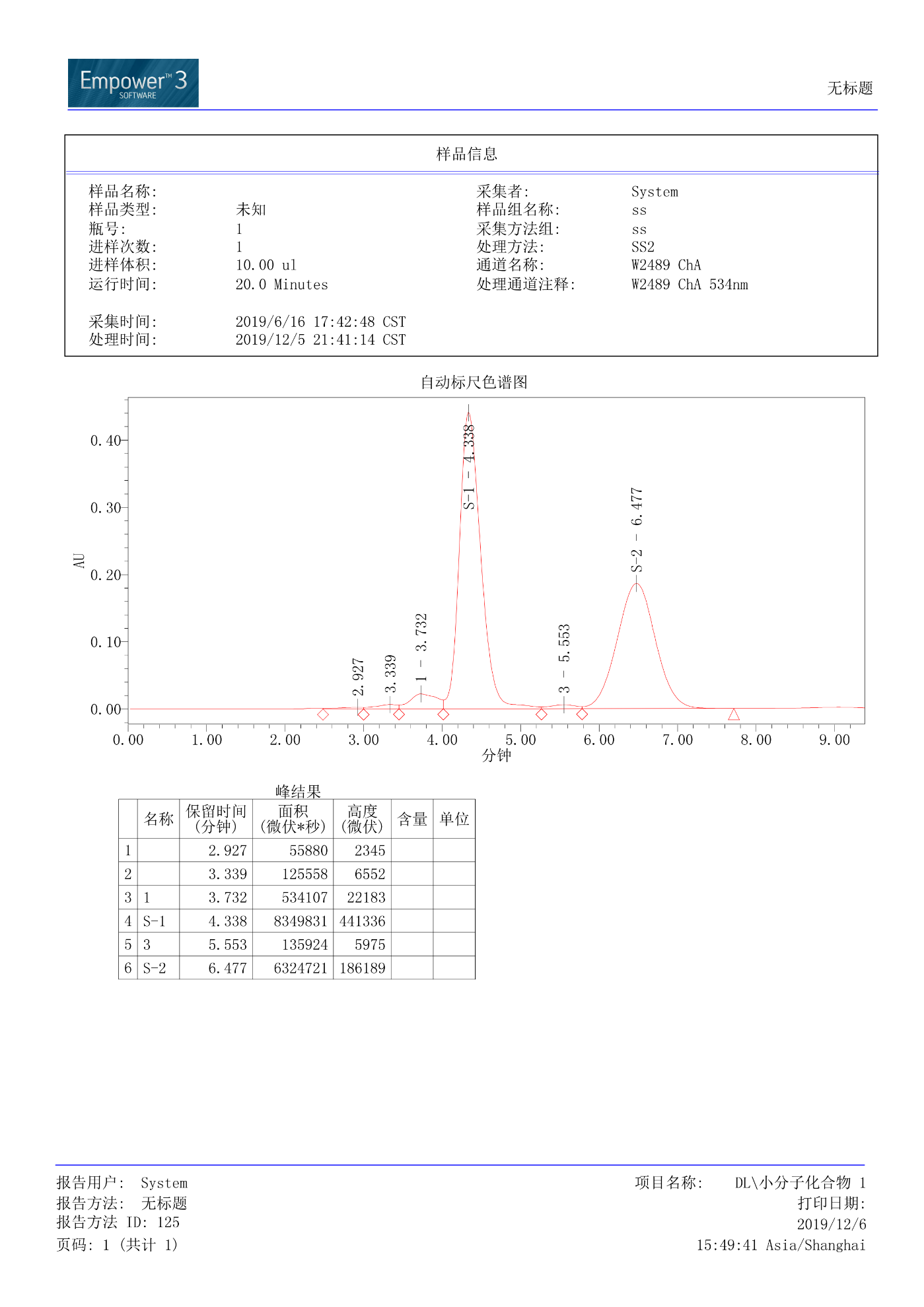


**Figure S2**. Liquid chromatography spectrometry of red pigments extracted from strain S2-4-1H^T^.

Two major peaks named S-1 and S-2 were indicated at the retention time of 4.338 min and 6.477 min, respectively. The other minor peaks were neglected in this study.


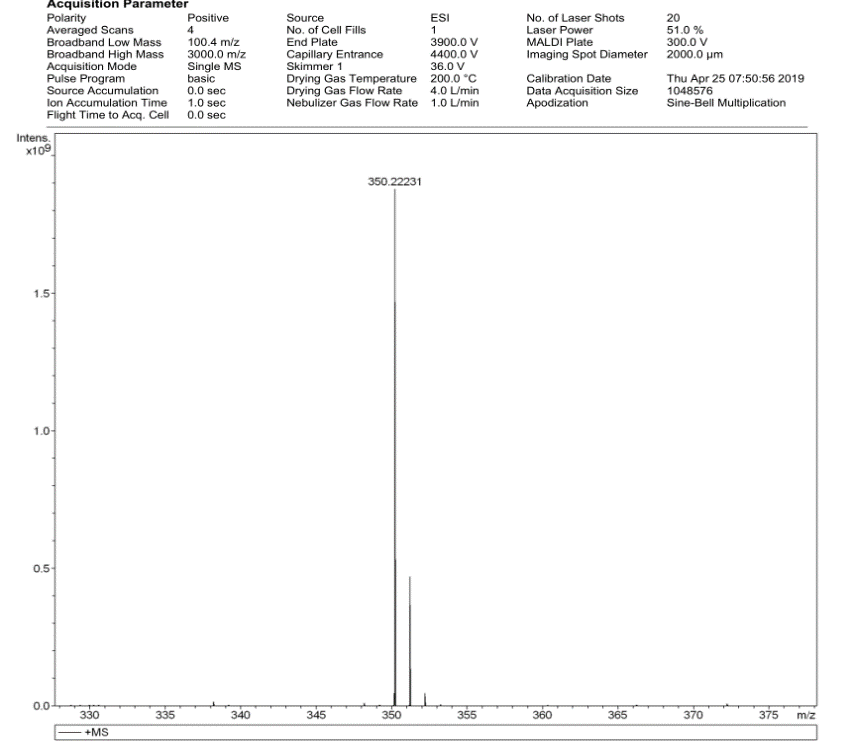

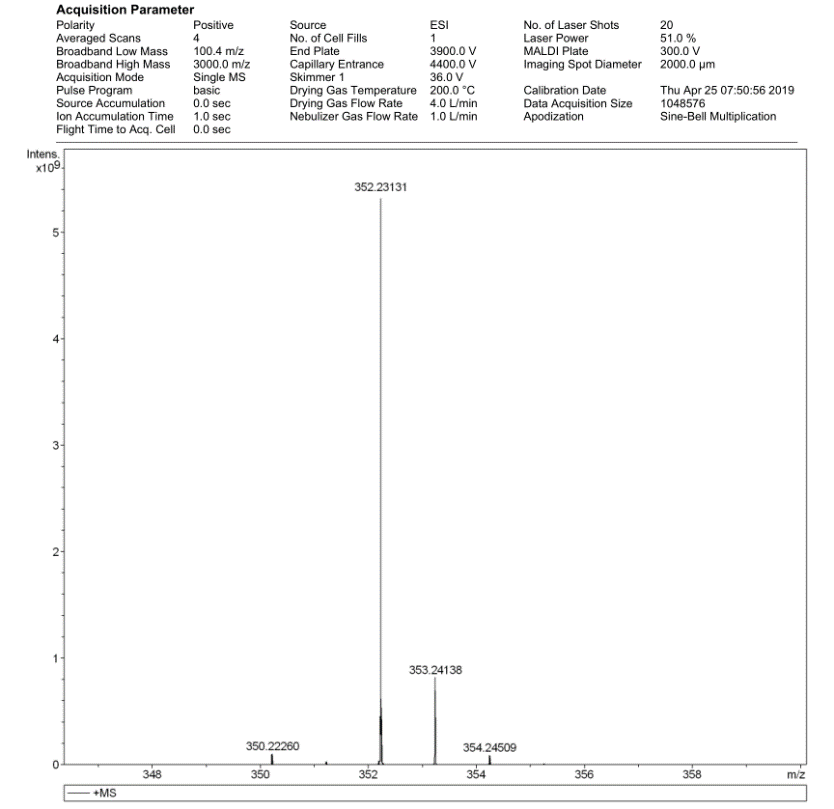


**Figure S3**. HRESIMS mass spectra of red pigments S-1 (left panel) and S-2 (right panel).


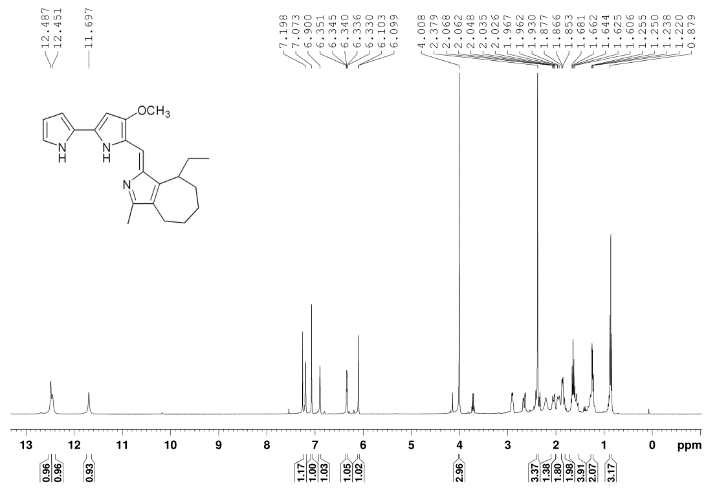

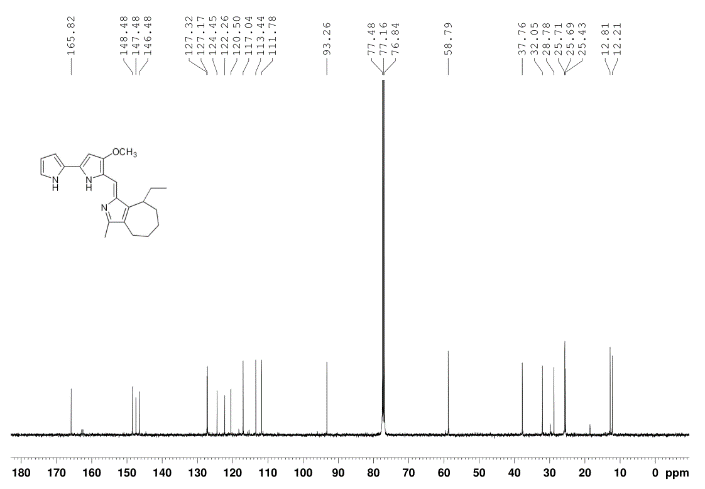


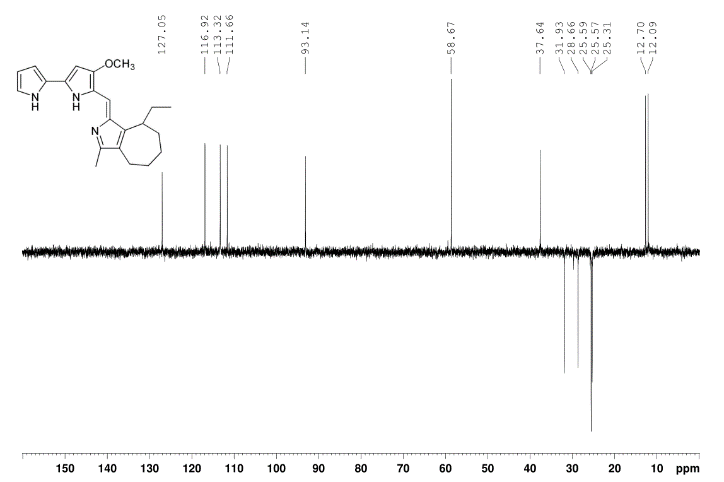

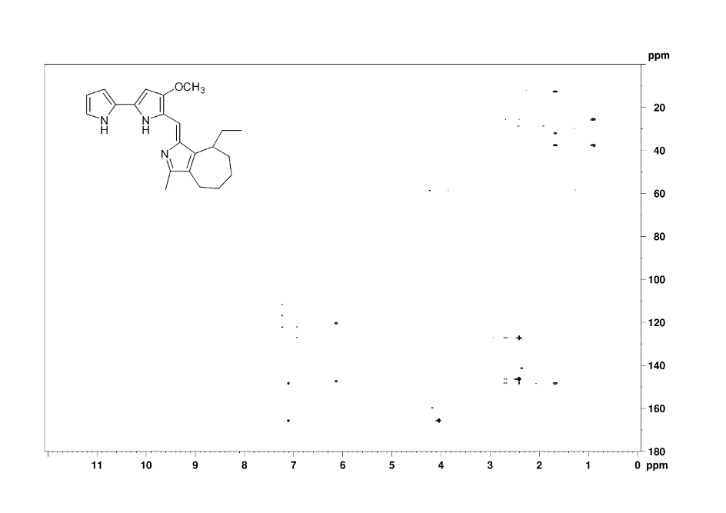


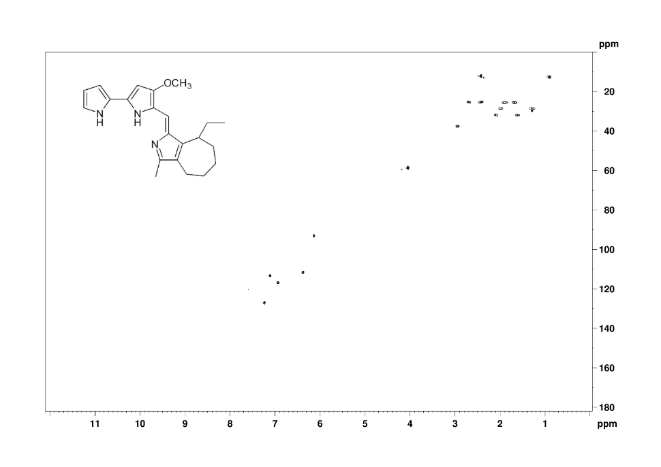


**Figure S4.** ^1^H-NMR, ^13^C-NMR, C-DEPT135, HMBC and HSQC of red pigment S-1 extracted and purified from strain S2-4-1H^T^.

^1^H-NMR spectrum (upper left panel); ^13^C-NMR spectrum (upper right panel); C-DEPT135 spectrum (middle left panel); Heteronuclear multiple bond correlation (HMBC) spectrum (middle right panel); Heteronuclear single quantum correlation (HSQC) spectrum (lower left panel).


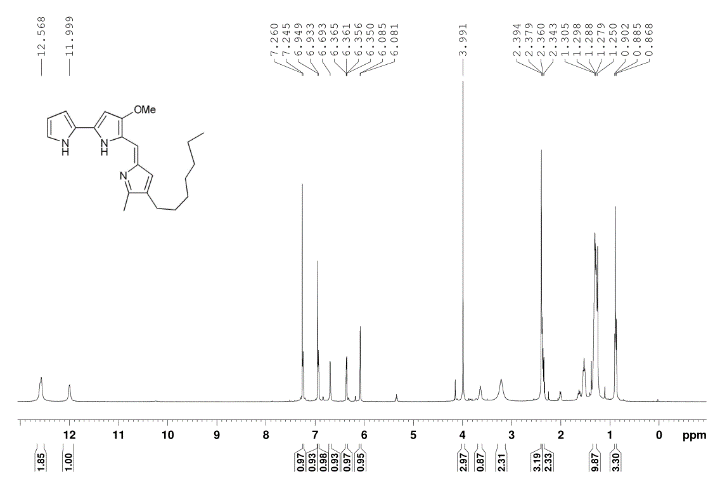

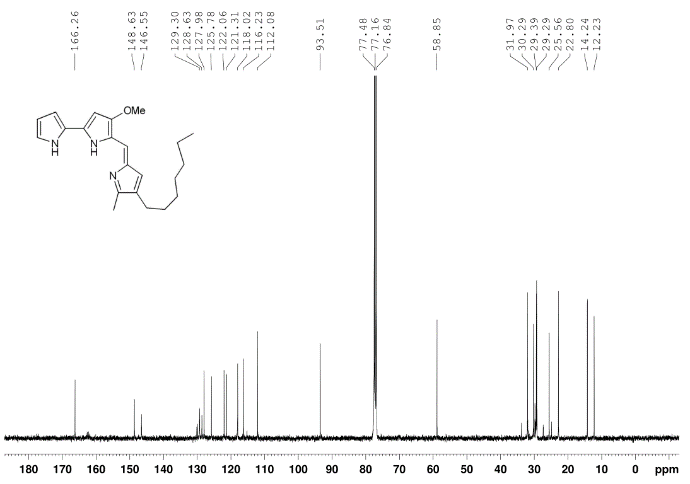


**Figure S5.** ^1^H-NMR (left panel) and ^13^C-NMR (right panel) of red pigment S-2 extracted and purified from strain S2-4-1H^T^.


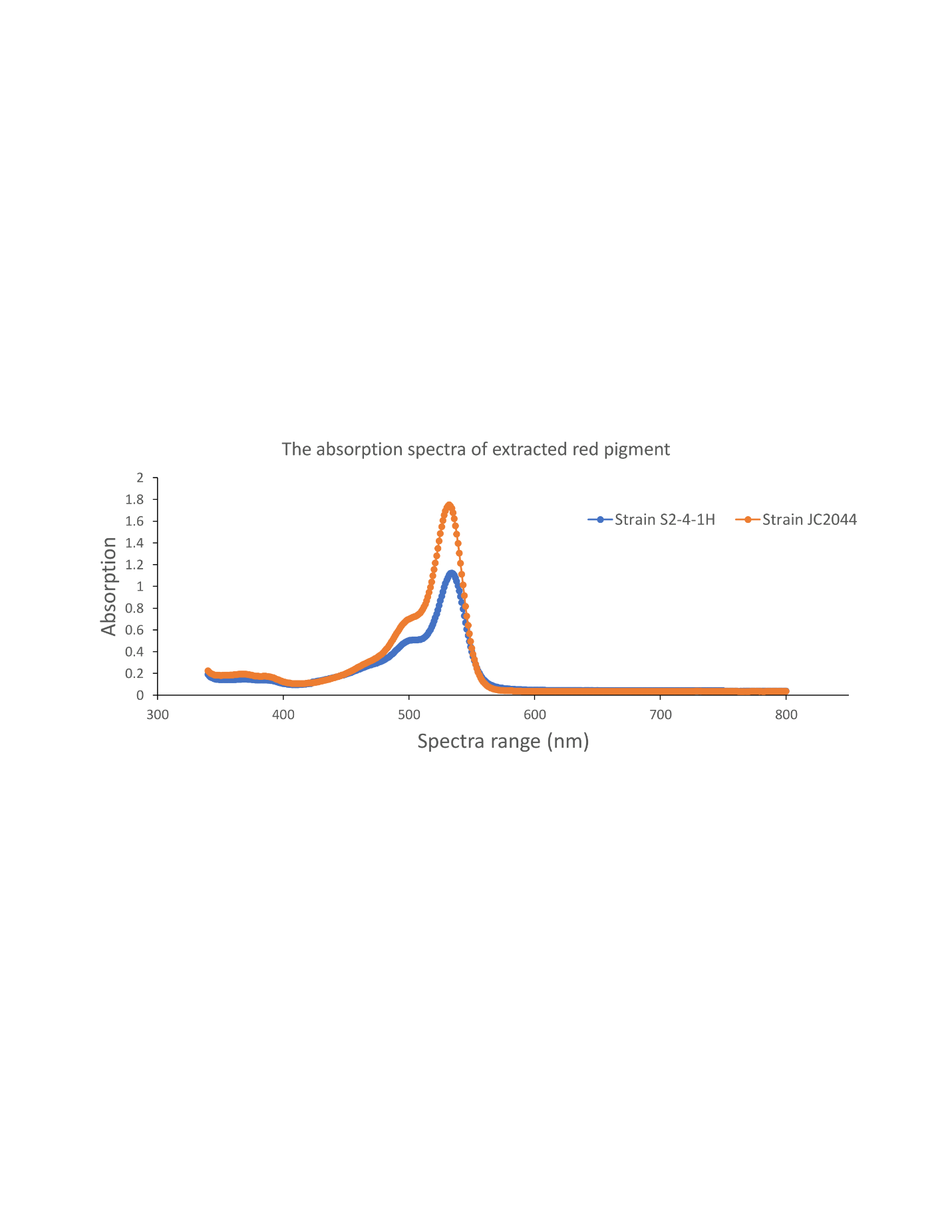


**Figure S6**. The absorption spectra of the pigment extracted from strain S2-4-1H^T^ and strain JC2044^T^.

*Pseudoalteronomas* were separated into two species-level clade, (1) represented *P. ruber*, and (2) represented *P. denitrificans*. Bar, 0.1 represented the amino acid substitution per position


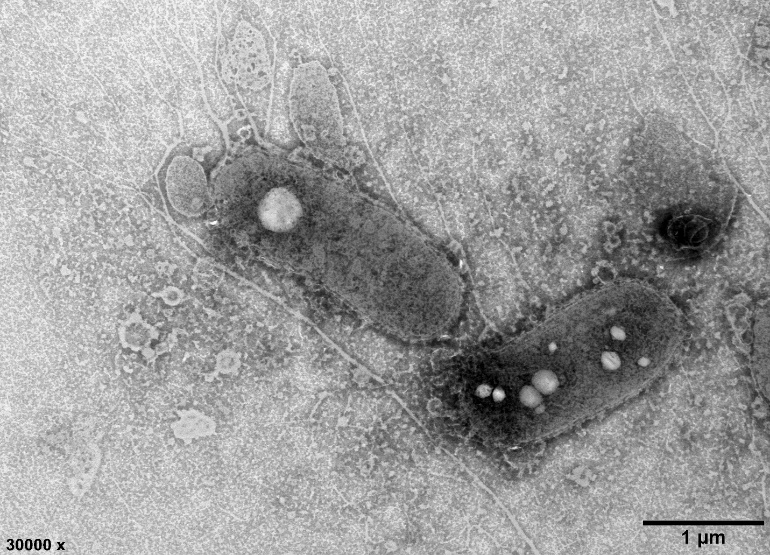


**Figure S7**. Transmission electron micrograph of negatively stained bacterial strain S2-4-1H^T^.


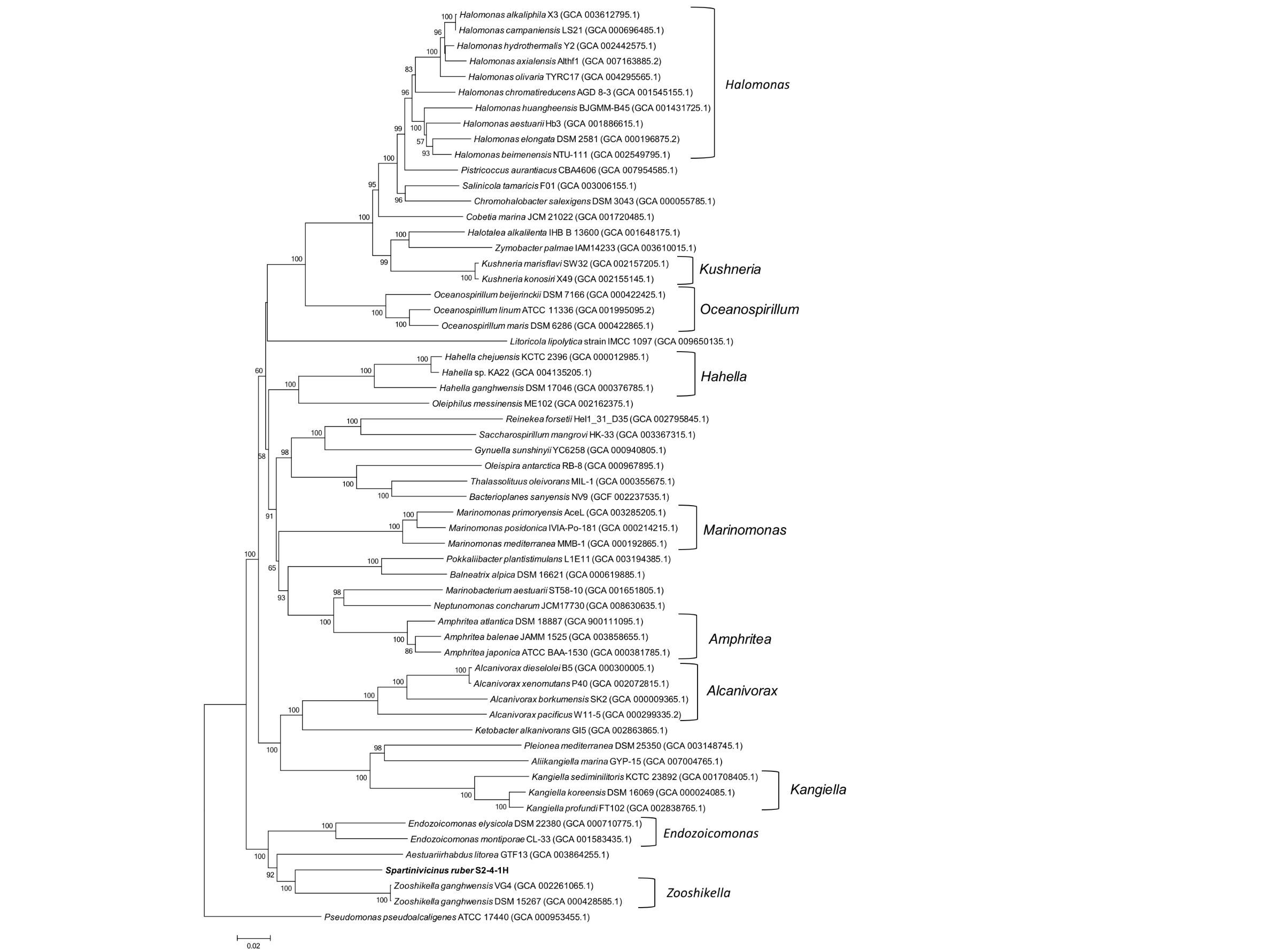


**Figure S8. Phylogenomic analysis of strain S2-4-1H^T^ compared with the members of the order *Oceanospirillales* based on whole genome sequences.**

Neighbor joining phylogenomic tree constructed based on 43 conserved marker genes consistent with the maximum likelihood phylogenetic analysis. Bootstrapping was carried out with 100 replicates to accommodate the computation for maximum likelihood inference (not shown). *Pseudomonas pseudoalcaligenes* ATCC 17440 (GCA_000953455.1) was selected as the outgroup. Bar, 0.02 represented the amino acid substitution per position.


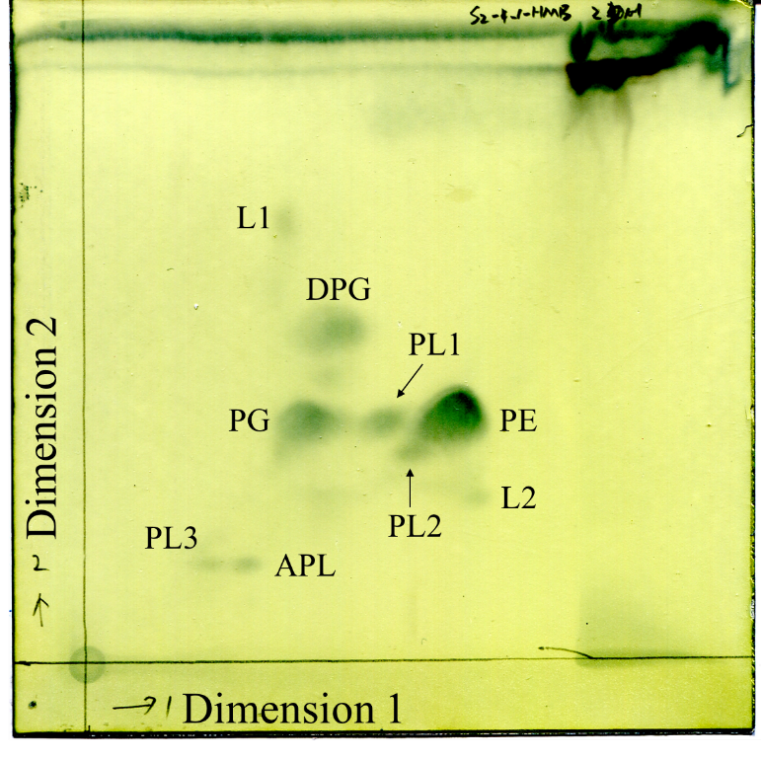


**Figure S9**. The polar lipids profiles of strain S2-4-1H^T^. Abbreviations: PE, phosphatidylethanolamine; PG, phosphatidylglycerol; DPG, diphosphatidylglycerol; PL, phospholipid; APL, unidentified aminophospholipid; L, unidentified polar lipid.
